# A systems biology approach to study non-alcoholic fatty liver (NAFL) in women with obesity

**DOI:** 10.1016/j.isci.2022.104828

**Published:** 2022-08-05

**Authors:** Abraham S. Meijnikman, Dimitra Lappa, Hilde Herrema, Omrum Aydin, Kimberly A. Krautkramer, Valentina Tremaroli, Louise E. Olofsson, Annika Lundqvist, Sjoerd Bruin, Yair Acherman, Joanne Verheij, Siv Hjorth, Victor E.A. Gerdes, Thue W. Schwartz, Albert K. Groen, Fredrik Bäckhed, Jens Nielsen, Max Nieuwdorp

**Affiliations:** 1 Departments of Internal and Experimental Vascular Medicine, Amsterdam University Medical Centers, Location AMC, Amsterdam, the Netherlands; 2 Department of Surgery, Spaarne Hospital, Hoofddorp, the Netherlands; 3 Systems and Synthetic Biology, Department of Biology and Biological Engineering, Chalmers University of Technology, Gothenburg, Sweden; 4 Wallenberg Laboratory, Department of Molecular and Clinical Medicine, Sahlgrenska Academy, University of Gothenburg, Gothenburg, Sweden; 5 Department of Pathology, UMC, University of Amsterdam, Cancer Center Amsterdam, Amsterdam, the Netherlands; 6 Laboratory for Molecular Pharmacology, Department of Neuroscience and Pharmacology, Faculty of Health Sciences, University of Copenhagen, Copenhagen, Denmark; 7 Novo Nordisk Foundation Center for Basic Metabolic Research, Faculty of Heath and Medical Sciences, University of Copenhagen, Copenhagen, Denmark; 8 Region Västra Götaland, Sahlgrenska University Hospital, Department of Clinical Physiology, Gothenburg, Sweden

**Keywords:** Biological sciences, Physiology, Human metabolism, Systems biology

## Abstract

Non-alcoholic fatty liver disease (NAFLD) is now the most frequent global chronic liver disease. Individuals with NAFLD exhibited an increased risk of all-cause mortality driven by extrahepatic cancers and liver and cardiovascular disease. Once the disease is established, women have a higher risk of disease progression and worse outcome. It is therefore critical to deepen the current knowledge on the pathophysiology of NAFLD in women. Here, we used a systems biology approach to investigate the contribution of different organs to this disease. We analyzed transcriptomics profiles of liver and adipose tissues, fecal metagenomes, and plasma metabolomes of 55 women with and without NAFLD. We observed differences in metabolites, expression of human genes, and gut microbial features between the groups and revealed that there is substantial crosstalk between these different omics sets. Multi-omics analysis of individuals with NAFLD may provide novel strategies to study the pathophysiology of NAFLD in humans.

## Highlights

• The gut microbiome is significantly different between women with and without NAFLD
• The NAFLD plasma metabolome is characterized by lipid and amino acids post prandial
• Women with NAFLD have distinct transcriptional profiles in liver and adipose tissue
• Women with NAFL have a different response upon MMT after massive weight loss

## Graphical abstract

**Figure:**
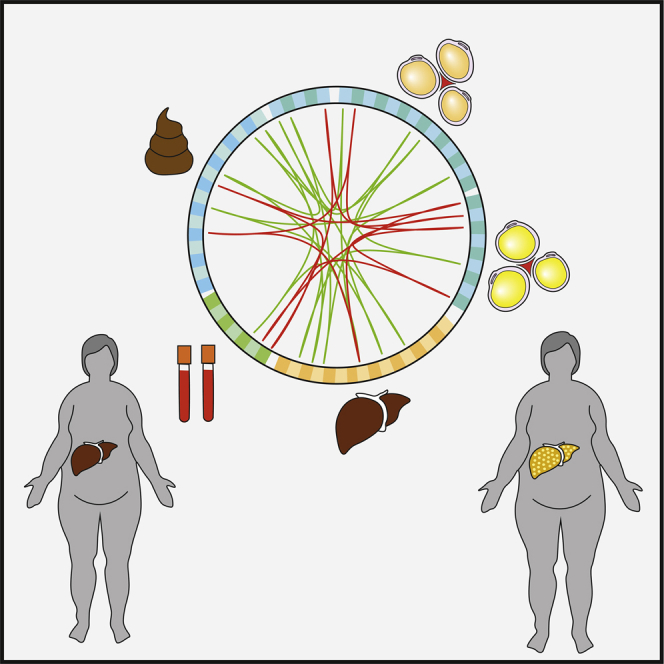

## Introduction

As a consequence of the pandemic spread of obesity, NAFLD is now recognized as the most prevalent chronic liver disease worldwide (Younossi et al., 2018). In the general population, one in four individuals is affected by NAFLD; this prevalence increases to over 80% in individuals with obesity (Younossi et al., 2018). NAFLD comprises a spectrum of clinical and histopathological abnormalities. These include simple steatosis and steatosis with mild inflammation (non-alcoholic fatty liver, NAFL) as well as steatosis with ballooning and inflammation (non-alcoholic steatohepatitis, NASH). Accumulation of fat in hepatocytes has long been considered a relatively benign condition. However, an estimated 30% of people with NAFL will develop NASH, a progressive form of liver disease that can lead to liver fibrosis, cirrhosis, and hepatocellular carcinoma (Farrell and Larter 2006). Advanced forms of NASH often require liver transplantation and are the main cause of liver-related deaths in NAFLD (Younossi et al., 2018). A recent report of a large nationwide cohort study investigating overall and cause-specific mortality in long-term follow-up of individuals with NAFLD, however, showed that individuals with NAFL also exhibited an increased risk of all-cause mortality driven by extrahepatic cancers and liver and cardiovascular disease (CVD) (Simon et al., 2020). Of concern, especially women with NAFLD are more susceptible to develop excess CVD events compared to age-matched men (Allen et al., 2019). In fact, NAFLD has a cardiovascular aging effect of approximately 18 years in women. Moreover, in general, women have a lower risk of developing NAFLD, but once the disease is established, women have a higher risk of disease progression (Balakrishnan et al., 2021). The rapidly growing prevalence of NAFLD and lack of effective treatment options to tackle this potentially debilitating disease will further increase obesity-related burden on public health and economies. In order to develop appropriate, sex-specific non-invasive diagnostic methods and treatment options, it is critical to deeply investigate the complex pathophysiology of NAFLD.

The underlying mechanisms that govern hepatic lipid accumulation and the predisposition to inflammation and fibrosis are complex and multifactorial, which is recapitulated in the multi-hit hypothesis that implicates that a myriad of factors are acting in a parallel and synergistic manner (Buzzetti et al. 2016). These factors include insulin resistance, adipocyte dysfunction, genetic variants, bile acid metabolism, the gut microbiome, and lipotoxicity (Aron-Wisnewsky et al., 2020; Marjot et al., 2020). The complexity of the contributing factors can mask different structural associations between metabolic activities in different tissues, prohibiting in-depth insight into molecular mechanisms underlying disease development. By applying a systems biology approach using multi-omics data, it is possible to deep phenotype individuals with or without metabolic diseases and, through data integration, identify the crosstalk between different relevant biological layers.

We here used a global approach to investigate factors that may contribute to NAFL development in women. Our systems biology approach allowed for integration of transcriptomics, metagenomics, and plasma metabolomics datasets from obese women with and without NAFL. Analyses of these integrated omics sets revealed a robust NAFL signature and highlight the additive value of a multi-omics approach to study NAFL pathophysiology.

## Results

To take a comprehensive approach to investigate factors that may contribute to NAFL development, we included individuals from our bariatric surgery cohort (the BARIA study) (van Olden et al., 2020), but excluded patients with type 2 diabetes mellitus (T2DM) to avoid confounding effects of long-term hyperglycemia or medication use. Since there are strong sex differences in hepatocellular and systemic processes in the pathophysiology and progression of NAFLD (Lonardo et al., 2019; Vandel et al., 2020), we focused on women. The study cohort comprised of 55 women for whom a multi-omics dataset was available, including fasting and two-hour post mixed meal test (MMT) plasma metabolome, liver and adipose tissue (subcutaneous and mesenteric) transcriptome, along with gut microbial metagenome. In addition, we analyzed the glucose and insulin response during the MMT before and one year after bariatric surgery to investigate differences in glucose metabolism between women with and without NAFL.

In total, 23 individuals (BMI 39.4 ± 3.0 kg/m^2^, age 45 ± 11 years) fulfilled the criteria for NAFL (biopsy-proven) whereas 32 individuals (BMI 40.2 ± 4.7 kg/m^2^, age 41 ± 10 years) had no NAFL (Table 1). NAFL ranged from grade 1 to grade 2 steatosis; none of our individuals had hepatocyte ballooning, a prerequisite for NASH diagnosis according to the SAF criteria (Bedossa et al., 2014). As expected, the ALT levels were increased in the NAFL group, whereas comorbidities such as insulin resistance (as assessed by MMT) and medication did not differ between groups, indicating a homogenous study population (Table 1, Figures S1 and S2).

**Table 1:** Baseline characteristics of the 55 women included

Characteristics	Non-NAFL = 32	NAFL = 23
**Demographic**		

Age *(years)*	41 ± 10	45 ± 11

**Anthropometric**		

BMI *(kg/m2)*	40.2 ± 4.7	39.4 ± 3.0
Type 2 diabetes mellitus *(n)*	0	0

**Clinical lab values (normal range)**		

ALP *(30–135 U/L)*	85 ± 21	84 ± 19
g-GT *(10 – 40 IU/L)*	26 (18–26)	28 (18–41)
ALT *(0–50 IU/L)*	25 (18–27)	36 (22–42)∗
AST *(0 – 35 IU/L)*	22 ± 4	26 ± 6
FPG *(<5.6 mmol/L)*	5.4 ± 0.5	5.6 ± 0.6
HbA1c *(<5.6%)*	5.4 ± 0.3	5.6 ± 0.2
HbA1c *(mmol/mol)*	35 ± 3	37 ± 2
Total cholesterol *(1.5 – 6.5 mmol/L)*	4.9 ± 1.1	4.9 ± 1.1
Triglycerides *(<1.7 mmol/L)*	1.4 (0.9–1.5)	1.7 (1.1–1.9)
HDL cholesterol *(≥1.0 mmol/L)*	1.3 ± 0.4	1.2 ± 0.3
LDL cholesterol *(< 3.0 mmol/L)*	3.1 ± 1.1	3.2 ± 0.8

**Histological parameters (number)**		

Steatosis grade score *(0,1,2,3)*	32,0,0,0	0,22,1,0
Lobular inflammation score *(0,1,2)*	14,17,1	0,21,2
Hepatocyte ballooning score *(0,1,2)*	32,0,0	23,0,0

Data is expressed as mean ± standard deviation or as median (interquartile range) depending on normality of the data. For histological scores, the number of individuals with a certain score is shown according to the Steatosis Activity and Fibrosis score (SAF).

NAFL, Non-Alcoholic Fatty Liver; BMI, body mass index; ALP, alkaline phosphatase; g-GT, gamma glutamyl transferase; ALT, alanine aminotransferase; AST, aspartate aminotransferase; FPG, fasting plasma glucose; HbA1c, Hemoglobin A1c; HDL, high-density lipoprotein; LDL, low-density lipoprotein.

∗indicate significant (p < 0.05) difference. Significance was calculated by either independent T test or Mann-Whitney U test depending on normality

### The gut microbial communities of individuals with and without NAFL significantly differ

**Figure 1:**
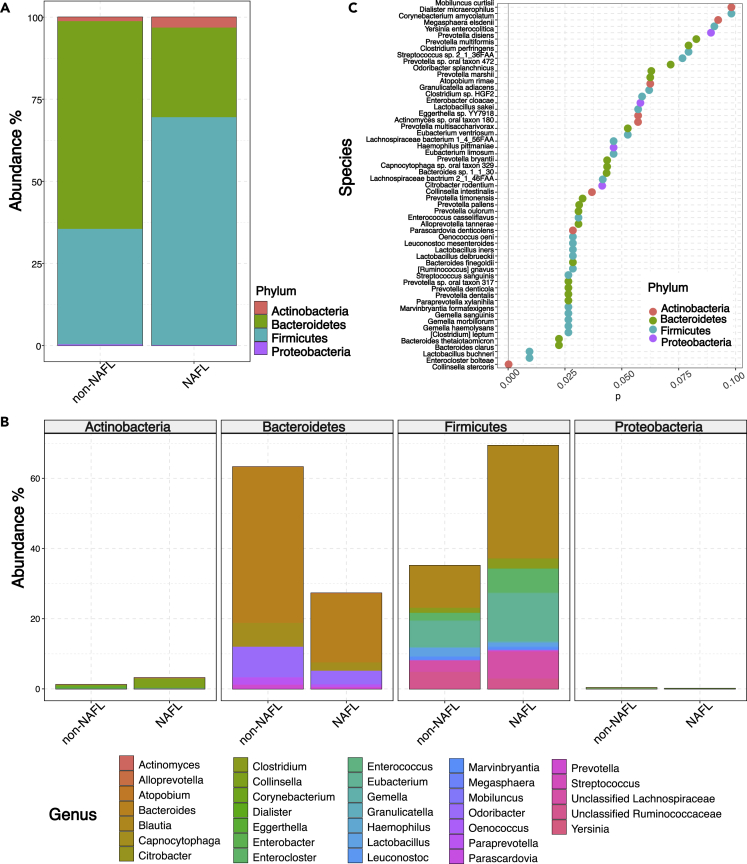
Microbial species and phyla between individuals with and without NAFL (A) Difference in total abundance of bacterial species indicated at the phylum level between individuals with and without NAFL. (B) Relative abundance and distribution within of differentially significant microbial species between individuals with and without NAFL. (C) 57 differentially significant microbial species between individuals with and without NAFL, after differential microbial species analysis with DESeq2 (adjusted p < 0.1) Likelihood Ratio Test for significance.

To characterize the gut microbiome in individuals with and without NAFL, we performed whole-genome shotgun sequencing of the fecal DNA and used MEDUSA to obtain taxonomic information (Karlsson et al. 2014). In order to assess if there is a difference in microbial alpha diversity between individuals with and without NAFL, we used a series of different metrics (Observed, Chao1, ACE, Shannon, Simpson, and Inverse Simpson). According to the alpha diversity metrics, the microbial diversity was similar in the two groups, which is in contrast to previous reports (Leung et al., 2016; Hoyles et al., 2018) (Figure S3). These previous observations analyzed individuals with a more progressive form of NAFLD (*i.e.,* NASH). In agreement with previous studies (Boursier et al., 2016; Loomba et al., 2017; Caussy et al., 2019), we observed that the microbiome was dominated by Firmicutes in individuals with NAFL, while Bacteroidetes was the most dominant phylum in individuals without NAFL (Figure 1A). We next assessed the bacterial species composition per individual (Figure S4). Even though we observed large inter-individual variation in the gut microbiome composition, PERMANOVA and beta dispersion analysis revealed that the two groups largely spatially overlap but have different centroids and different dispersions (Figure S5). In total, 57 bacterial species differed significantly between individuals with and without NAFL (Figure 1A). Three bacterial species were at least twice as abundant in individuals with NAFL (Table S1). One of these species belonged to the phylum Actinobacteria (*Collinsela stercoris*) whereas two belonged to Firmicutes (*Lactobacillus buchneri* and *Lactobacillus iners*). In individuals without NAFL, 11 bacterial species were at least twice as abundant compared to individuals with NAFL. Of these 11 bacterial species, six belonged to the phylum Bacteroidetes (*Prevotella oulorum*, *Prevotella sp. oral taxon 317*, *Prevotella sp. Oral taxon 472*, *Prevotella multisaccharivorax*, *Prevotella dentalis*, and *Prevotella bryantii*), two belonged to Firmicutes (*Lactobacillus delbrueckii* and *Enterococcus casseliflavus*), and three belonged to Proteobacteria (*Citrobacter rodentium*, *Yersinia enterocolitica*, and *Haemophilus pittmaniae*). In summary, even though alpha diversity did not differ significantly between the two groups, 57 bacterial species differed significantly and mainly belonged to the Bacteroidetes and Firmicutes phylum.

### The NAFL-associated metabolome is characterized by increased lipid and amino acids in postprandial conditions

**Figure 2:**
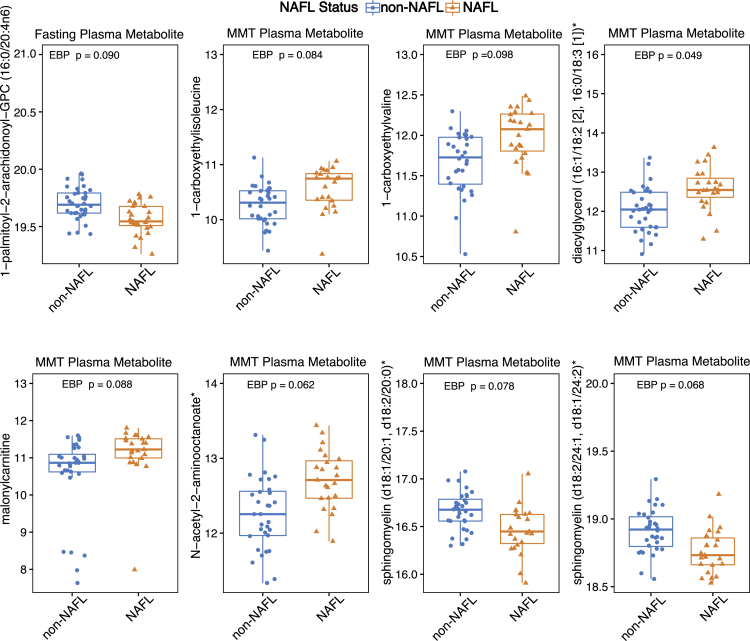
Log scale abundance of differentially significant metabolites between individuals with and without NAFL in fasting and postprandial plasma metabolomics Differential metabolite analysis was conducted with the HybridMtest package and p-adjusted based on Estimated Bayesian Probability (p < 0.1).

Since microbiome-associated factors such as microbial metabolites are more and more recognized as disease-modifying factors, including in NAFL development (Aron-Wisnewsky et al., 2020), we performed plasma metabolomics analyses on fasting and post MTT samples to reveal metabolite-based phenotypes of NAFL. Out of 988 metabolites, phosphathidylcholine 1-palmitoyl-2-arachidonoyl-GPC (16:0/20:4n6) was the only significantly altered metabolite in fasted individuals and was lower in individuals with NAFL (Figure 2, Table S2). Since humans rarely reside in a fasting state for a long period of time, the liver is continuously exposed to nutrients and (microbial) metabolites from the intestine. Thus, postprandial plasma samples might provide a more representative view on circulating metabolites in individuals with NAFL. Indeed, seven metabolites differed significantly in the postprandial state. Five metabolites were more abundant in NAFL and two were more abundant in non-NAFL individuals (Figure 2, Table S3). Two sphingomyelin metabolites were decreased in individuals with NAFL whereas diacylglycerol, a signaling lipid previously linked to hepatic insulin resistance and NAFLD (Samuel and Shulman 2018), was more abundant in individuals with NAFL. In agreement with previous studies demonstrating that circulating amino acids are increased in individuals with NAFL (Kalhan et al., 2011; Gaggini et al., 2018), the branched-chain amino acids (BCAA) derivatives 1-carboxyethylisoleucine and 1-carboxyethylvaline were increased in individuals with NAFL. Alterations in amino acids in cardiometabolic disease have been tightly linked to insulin resistance. Since both insulin and glucose levels did not differ during the MMT (Figure S2), our data suggest that these alterations may be independent of altered glucose metabolism.

### Distinct transcriptional profiles in liver, subcutaneous, and mesenteric adipose tissue

Since several studies have demonstrated that (microbial) metabolites exert metabolic actions on distal tissues and organs (Krautkramer et al. 2020), we profiled hepatic, mesenteric, and subcutaneous adipose tissue transcriptomes to improve our understanding of the interrelation between alterations in the plasma metabolome and gene expression. By using DESeq2 (Love et al. 2014), we identified differently expressed genes between individuals with and without NAFL. Analyses of the hepatic transcriptome identified 52 genes that were differently expressed between individuals with and without NAFL. Of these genes, 13 were upregulated and 39 were downregulated in individuals with NAFL compared to individuals without NAFL (Table S4). KEGG pathway enrichment analysis using EnrichR (Chen et al., 2013) identified that pathways involved in several cancers were enriched in individuals with NAFL, which may indicate increased cell proliferation. Furthermore, the hypoxia-inducible factor 1 (HIF-1) signaling pathway, which has previously been linked to NAFLD pathogenesis (Semenza 2007), was enriched in the liver of individuals with NAFL. The only significant pathway that was enriched in individuals without NAFL was the pathway involved in arginine and proline metabolism (Table 2). Since adipose tissue and the liver communicate with each other (Azzu et al., 2020), we next investigated the transcriptome of two different adipose tissue depots. In subcutaneous adipose tissue, 19 genes were significantly different between the groups of which 15 were higher in individuals with NAFL and four were higher in individuals without NAFL (Table S5). The mesenteric adipose tissue transcriptome revealed that 56 genes differed significantly between individuals with and without NAFL. Of these, 34 genes were upregulated and 22 genes were downregulated in individuals with NAFL compared to individuals without NAFL (Table S6).

**Table 2:** KEGG metabolic pathways up- or downregulated in individuals with and without NAFL

Tissue	Regulation	Pathway	P-value
Liver	Upregulated in NAFL	HIF-1 signaling pathway	0.0019
		Bladder cancer	0.026
		Endometrial cancer	0.037
		Central carbon metabolism in cancer	0.041
		Non-small-cell lung cancer	0.042
		Arginine and proline metabolism	0.089
	Downregulated in NAFL	Pyrimidine metabolism	0.103
		Cortisol synthesis and secretion	0.116
		Bile secretion	0.128
		Drug metabolism	0.186
Mesenteric adipose tissue	Upregulated in NAFL	Galactose metabolism	2.119E-7
		Carbohydrate digestion and absorption	5.6684E-5
		Protein digestion and absorption	4.767E-4
		Starch and sucrose metabolism	0.002
		Fat digestion and absorption	0.002
	Downregulated in NAFL	Prion diseases	0.036
		Legionellosis	0.056
		Complement and coagulation cascades	0.080
		Systemic lupus erythematosus	0.1308
		Herpes simplex virus 1 infection	0.407
Subcutaneous adipose tissue	Upregulated in NAFL	IL-17 signaling pathway	4.253E-5
		AGE-RAGE signaling pathway in diabetic complications	5.283E-5
		TNF signaling pathway	7.019E-5
		Prion diseases	3.079E-4
		African trypanosomiasis	3.444E-4
	Downregulated in NAFL	Regulation of response to oxidative stress	0.002
		Regulation of response to stress	0.002
		Positive regulation of G2/M transition of mitotic cell cycle	0.003
		Positive regulation of cell cycle G2/M phase transition	0.003
		Positive regulation of peptidyl-threonine phosphorylation	0.005

According to KEGG pathway analysis, interleukin (IL)-17, advanced glycation end products (AGE), and tumor necrosis factor (TNF),signaling pathways were enriched in individuals with NAFL in subcutaneous adipose tissue, whereas response to oxidative stress was not enriched underscoring the well-established link between adipose tissue inflammation and NAFL (Arab et al. 2018) (Table 2). In mesenteric adipose tissue, carbohydrate, galactose, sucrose, and protein metabolism pathways were enriched in mesenteric adipose tissue from individuals with NAFL, while pathways involved in infectious disease were not enriched (Table 2). Furthermore, pathways associated with fat digestion and absorption were enriched in individuals with NAFL. This further strengthens the link between alterations in diacylglycerol and adipose tissue dysfunction. Transcriptome analyses from all three tissues showed distinct differences in gene expression and pathways relevant for the development of NAFL such as HIF-1 signaling, inflammation, and fat digestion and absorption.

### Multi-omics integration creates a signature for NAFL

**Figure 3:**
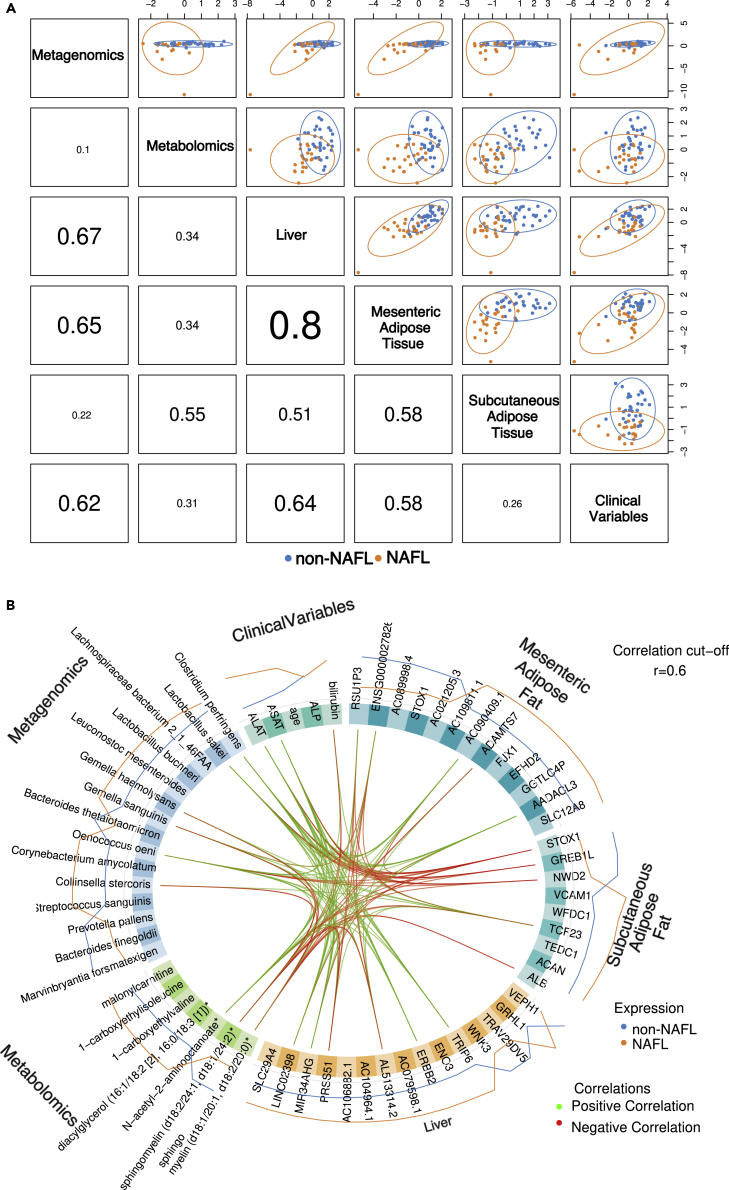
DIABLO analysis and correlations among multi-omics datasets for individuals with and without NAFL (A) Total correlation matrix for all the different omic datasets after Sparse Partial Least Squares Regression with mixOmix DIABLO. Highest correlation is observed for genes from liver and mesenteric adipose tissue. (B) Circular correlation plot by Data Integration Analysis for Biomarker discovery using a Latent cOmponents (mixOmics DIABLO), for top contributing components to from each omics dataset (metabolites, genes, and bacterial species). Correlation cut-off is r = 0.6. Signature involves Prevotella species, branched-chain amino acid metabolites, sphingolipid metabolites, diacyglycerols, liver genes highly involved in cancer pathways, renin-angiotensin system, mesenteric adipose tissue genes involved in carbohydrate metabolism and subcutaneous adipose tissue genes involved in mitochondrial translation/elongation.

The individual omics datasets thus far revealed differences in the fecal metagenome, the plasma metabolome, and tissue transcriptome between the groups. However, discriminative, analyses of these individual omics sets do not provide insight in the interrelation between the different biological layers. We therefore constructed a multivariate model to identify crosstalk events between these different tissues and metagenomic, metabolomic, and clinical datasets by fitting a sparse partial least squares discriminant analysis with Data Integration Analysis for Biomarker discovery using Latent cOmponents (DIABLO) (Singh et al., 2019). DIABLO simultaneously calculates the correlations between all input omics datasets and selects a minimal set of input variables that differentiate between individuals with and without NAFL. This approach revealed correlations between the different tissues. The correlation between liver and mesenteric adipose tissue transcriptomics particularly stands out (r = 0.8), followed by liver transcriptomics and the fecal metagenome (r = 0.67; Figure 3A).

In addition, the full correlation matrix revealed the interrelation between particular metabolites, bacterial species, and genes that can be used to generate biological hypotheses that can be used to further unravel the pathophysiology or develop next-generation therapeutic strategies for NAFL (Figure 3B). For example, N-acetyl-2-aminooctanoate, *Lactobacillus sakei*, hepatic *TRIP6* (Thyroid Hormone Receptor Interactor 6), *ERBB2* (Erb-B2 Receptor Tyrosine Kinase 2), and *MIR34AHG* (*MIR34A* Host Gene affiliated with the lncRNA class) were all upregulated in NAFL and correlated positively with each other (r ≥ 0.6), suggesting that this metabolite could be of bacterial origin, or that the circulating levels are influenced by the gut microbiome. Moreover, the correlation between this metabolite and *TRIP6* and *ERBB2,* two genes that were recently identified to play a role in the pathophysiology of NAFLD (Machado et al., 2015; Wang et al., 2016), suggests that upregulation of these genes can be induced via circulating metabolites. In addition, N-acetyl-2-aminooctanoate was positively correlated with *AADACL* (Arylacetamide Deacetylase-Like 3) in mesenteric adipose tissue, which is a gene involved in lipolysis of adipose tissue and thus contributes to hepatic triglyceride accumulation (Quiroga and Lehner 2018). To which extent these genes are regulated by bacterial strains or metabolites need to be further investigated. 1-carboxyethylvaline was positively correlated with *ACAN* in subcutaneous adipose tissue. Furthermore, diacylglycerol was positively correlated with *WFDC1* and *ACAN* in subcutaneous adipose tissue. *WFDC1* and *ACAN* in the subcutaneous adipose tissue were highly enriched in gene sets involved in mitochondrial translation/elongation, suggesting a strong association between BCAAs and potential regulative signaling from adipose tissue.

**Figure 4:**
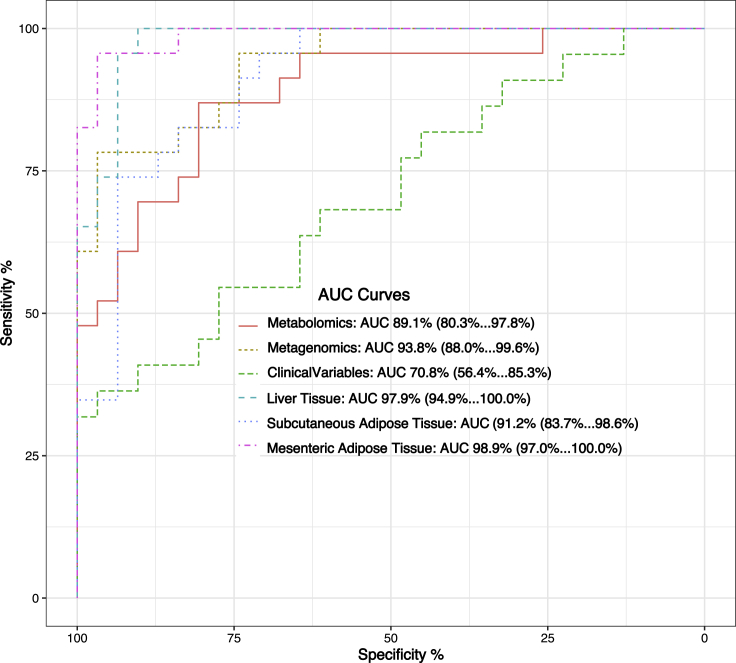
AUC predictive capacity for each omic dataset from DIABLO analysis All the transcriptomics datasets and the chosen genes can very accurately predict NAFL. Both DIABLO chosen Metabolome and Metagenome datasets outperform the Clinical variables in NAFL predictive capacity, with AUC = 89.1% and 93.8%, respectively, versus AUC = 70.8%.

Finally, to quantify the robustness of the individual omics signatures obtained by the integrative analysis, the power of every chosen omics subset by DIABLO to predict NAFL was assessed (Figure 4). A series of generalized linear models (GLMs) aimed to investigate whether the minimal discriminatory signal of the omics could outperform the clinical variables capacity to correctly predict NAFL. As anticipated, the performance of the signature found in the liver transcriptome was very high with an area under the curve of 0.98, followed by the visceral adipose tissue and subcutaneous adipose tissue. The postprandial metabolites and gut microbial species signatures appear to be more accurate prognostic markers of NAFL when compared to the chosen clinical variables.

To differentiate if these signatures are driven by hepatic steatosis (the prerequisite for NAFLD diagnoses) or lobular inflammation, we performed the same analyses but then between women with (n = 41) and without lobular inflammation (n = 14) and observed no distinct differences between al the omics sets suggesting that these signatures are driven mainly by the steatosis component (data now shown).

In summary, the computational framework used here for integrating various omics datasets successfully identified a highly correlated discriminatory signature for NAFL that included BCAA metabolites, diacylglycerol, liver genes involved in HIF-1 signaling, mesenteric adipose tissue genes involved in fat metabolism, and subcutaneous adipose tissue genes that are part of mitochondrial translation/elongation.

### Women with NAFL have a different response upon MMT after massive weight loss

**Figure 5:**
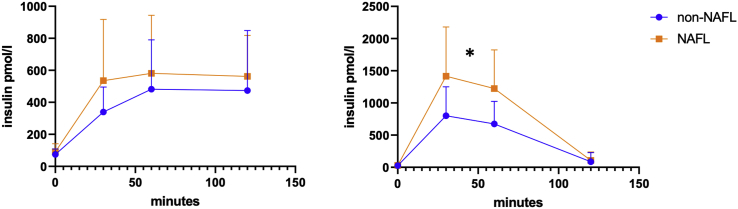
Insulin excursions during the mixed meal test in women with and without NAFL before (A) and one year after bariatric surgery (B)

To further substantiate that the alterations have clinical relevance, we analyzed the mixed meal data one year after bariatric surgery. Interestingly, at baseline we did not observe significant differences between individuals with and without NAFLD in glucose and insulin response during the MMT, but one year after bariatric surgery and massive weight loss, a clear difference was observed between the two groups in insulin but not in glucose during the MMT, which was significant (Figure 5). These results further suggest that whole body metabolism is indeed different in this early phase of the disease. Given the fact that weight loss and decrease in liver transaminases were not different, these data suggest that these differences are due to the inherent differences in whole body metabolism.

## Discussion

Here, we used a systems biology approach to identify factors that may contribute to NAFL development by analyzing six omics datasets of 55 women—who only differed in the presence of hepatic steatosis—including fecal metagenomics, plasma metabolomics, and liver, subcutaneous, and mesenteric adipose tissue transcriptomics. NAFLD is a multifactorial disease, which is underscored in the present study by showing that in each individual omics dataset, differences between women with and without NAFL could be observed. Suggesting that whole body metabolism is already altered in this early stage of the disease. The alterations in gut microbial composition are in line with previous work conducted by other independent groups showing that in subjects with NAFL, the gut microbiome is dominated by members of Firmicutes (Boursier et al., 2016; Loomba et al., 2017). However, our findings are in contrast with a recent report where liver steatosis was anticorrelated with Firmicutes (Hoyles et al., 2018). Nevertheless, it is plausible that there is not one unique microbiome signature for NAFLD, bearing in mind that the human microbiome is shaped by multiple factors such as age, sex, and disease state (Meijnikman et al., 2018). On species level, we observed a decrease in *Prevotella* species in individuals with NAFL. Interestingly, most of the *Prevotella* species were of oral origin, which is in contrast to previous findings (Atarashi et al., 2017). However, the mechanism and clinical significance underlying the increased transfer of oral bacteria to the gut remain to be elucidated. Subtle changes in the plasma metabolome were observed, especially in the post MMT samples, emphasizing that early changes in metabolism are more pronounced post meal than in fasting conditions. Alterations in BCAA composition in individuals with cardiometabolic disease are often explained to be caused by impaired amino acid metabolism linked to insulin resistance in the liver or muscle (White and Newgard 2019). Since insulin and glucose levels did not differ during the MMT, this indicates that these changes are independent of insulin resistance and opens up the prospect that these changes have been derived from another origin, potentially the gut microbiome (Krautkramer et al., 2020). Diacylglycerol, which is associated with NAFLD (Samuel and Shulman 2018), was increased in post MMT plasma of individuals with NAFL. Diacylglycerol is synthesized intracellularly from specific lipid precursors such as phosphatidylcholines, possibly including the metabolite that was increased in fasting conditions in individuals with NAFL.

To further investigate the relation between alterations in microbial composition and metabolites in host metabolism, we analyzed the transcriptome of liver and two adipose tissue depots obtained during surgery. Pathways previously suggested to play a pivotal role in the development of NAFLD such as the HIF-1 signaling pathway in the liver, fat and glucose metabolism, and inflammation in adipose tissue were increased in individual with NAFL. Nevertheless, the exact mechanisms that contribute to these pathways, especially in early disease, remain largely unknown. Therefore, we constructed a multivariable model to objectively quantify the crosstalk between these different omics datasets. We observed strong correlations between omics datasets, especially between mesenteric adipose tissue and liver transcriptomic data (r = 0.8) and between liver and subcutaneous adipose tissue (r = 0.51). These observations are in line with the current concept that adipocyte dysfunction plays a pivotal role in the pathophysiology of NAFLD (du Plessis et al., 2015; Arab et al., 2018). Adipose tissue expansion of both the subcutaneous and the visceral compartment leads to hypoxia-induced hypersecretion of adipocytokines such as tumor necrosis factor (TNF) and interleukin (IL) 6 by the adipocytes as well as by the inflammatory immune cells that accumulate in adipose tissue of individuals with obesity (Fernandez-Real et al., 2001; du Plessis et al., 2015). When reaching the liver through the portal vein, these mediators, together with increased levels of lipid metabolites such as diacylglycerols observed during metabolic dysregulation, can contribute to the development and progression of NAFLD (Arab et al., 2018). Interestingly, KEGG pathway enrichment of the differential significant genes of both mesenteric and subcutaneous adipose tissue revealed that pathways involved in fat and glucose metabolism and TNF signaling were upregulated in NAFL, respectively, underscoring the potential role of the adipose tissue in the development of NAFLD.

Recently, it was shown that there is a considerable link between the liver, the gut microbiome, and gut microbial metabolites (Hoyles et al., 2018). In this study, postprandial metabolomes and fecal metagenomes in general did not correlate with each other. A more in-depth view, however, revealed associations among metabolites belonging to amino acid metabolism, bacterial species, and liver genes. For example, N-acetyl-2-aminooctanoate, *Lactobacillus sakei*, *and* hepatic *TRIP6*, *ERBB2*, and *MIR34A* were all upregulated in NAFL and correlated positively with each other (r ≥ 0.6). Of interest, *TRIP6*, is an upstream activator of the transcriptional co-activators YAP (or YAP1) and TAZ and are involved in the pathogenesis of NAFLD (Machado et al., 2015; Wang et al., 2016). Also, genes involved in the hippo-signaling pathway were associated with this metabolite and bacterial strains. Hippo-signaling and downstream effectors are involved in a multitude of cell and non-cell autonomous functions including metabolism, cell proliferation, and survival (Machado et al., 2015). Interestingly, in the total correlation matrix, non-coding RNAs (*LINC02398* and *MIR34AHG*) and clone (*AC106882.1; AC109811.1*) of liver and mesenteric adipose fat were included. To what extent these non-coding and clones are associated with transcriptional regulation and are involved in the pathogenesis of NAFL remain to be investigated. Although our results are of associative nature, DIABLO full matrix correlation highlights the interrelation between metabolites, bacterial species, and genes and can be used to generate hypothesis to further study the pathophysiology of NAFL in humans.

In conclusion, our study provides a comprehensive multi-omics analysis of women with NAFL, providing a different strategy to study the pathophysiology of NAFL in women. Even though it is increasingly recognized that NAFL, also referred as “simple steatosis”, is more than just the passive accumulation of excessive fat, we further emphasize this by showing differences in metabolites, genes, and gut microbial species between individuals with and without NAFL. This is important work considering the fact that women with NAFLD have a higher change of CVD events, mortality, and disease progressions, even in the absence of severe hepatic inflammation and scarring (Allen et al., 2019). To what extent these findings are related to the severe outcome in women remain to be investigated. Lastly, by building a multivariate model, we revealed that there is substantial crosstalk between these different omics sets. Our model suggests that in early stages of the disease, adipocyte dysfunction is the predominant factor in disease development followed by gut microbial composition and plasma metabolites.

### Limitations of the study

We note that the analyses of human omics datasets in our study have some limitations. Here, we used tissue and plasma samples obtained from women who underwent bariatric surgery, which may introduce relevant biases in particular pre-operative weight loss with a subsequent decrease in liver volume. However, individuals who had lost more than 3% of weight in the month prior to surgery or more than 5% six months before surgery were excluded. We therefore ensure that the samples were obtained in a relatively stable period. The relatively low number of individuals (n = 55) in this study could potentially introduce bias to this particular modeling approach, especially since we did not have a validation cohort available to confirm these signatures. Therefore, external validation of these metabolites is warranted or should be further evaluated. Nevertheless, it is considerably challenging to come across similar multi-omics datasets in an external cohort, postprandial metabolome in particular, that include the same metabolites. Another limitation is that with the current study design we were not able to investigate to what extent the robust NAFL signature in each omics set contribute to the increased risk of developing CVD or adverse clinical outcome.

## STAR★Methods

### Key resources table

**Table:** 

REAGENT or RESOURCE	SOURCE	IDENTIFIER
**Biological samples**		

Human fecal metagenomics data	BARIA cohort (PI prof M. Nieuwdorp)	(van Olden et al., 2020)
Human liver RNA sequencing data	BARIA cohort (PI prof M. Nieuwdorp)	(van Olden et al., 2020)
Human subcutaneous adipose tissue sequencing data	BARIA cohort (PI prof M. Nieuwdorp)	(van Olden et al., 2020)
Human visceral adipose tissue sequencing data	BARIA cohort (PI prof M. Nieuwdorp)	(van Olden et al., 2020)
Human plasma metabolomics data	BARIA cohort (PI prof M. Nieuwdorp)	(van Olden et al., 2020)

**Deposited data**		

Liver and adipose tissue transcriptomics	European Nucleotide Archive	ENA PRJEB47902
Fecal metagenomics	European Genome-Phenome Archive	EGAS00001005704

**Software and algorithms**		

MEDUSA pipeline	n/a	(Karlsson et al., 2014)
Bowtie2	n/a	(Langmead and Salzberg 2012)
DESeq2	n/a	https://bioconductor.org/packages/release/bioc/html/DESeq2.html
Phyloseq	n/a	https://bioconductor.org/packages/release/bioc/html/phyloseq.html
DIABLO	n/a	http://mixomics.org/mixdiablo/

**Other**		

HiSeq instrument	Illumina	N/A
DNA extraction kit	QIAamp DNA Mini kit	N/A

### Resource availability

#### Lead contact

Further information should be directed to and will be fulfilled by the lead contact prof. dr. Max Nieuwdorp (m.nieuwdorp@amsterdamumc.nl)

#### Materials availability

This study did not generate new unique reagents.

### Experimental model and subject details

#### Ethical approvals and patients clinical information

The recruitment of participants was conducted from the BARIA study (van Olden et al., 2020)with a total of 55 individuals included. The baseline characteristics of these participants are described in Table 1. The study was performed in accordance with the Declaration of Helsinki and was approved by the Academic Medical Center Ethics Committee of the Amsterdam UMC (Trialregister: BARIA study NL8983). All participants provided written informed consent.

### Method details

#### Material collection

Individuals underwent a complete metabolic work-up at the start of their bariatric surgery trajectory. Anthropometric measurements including height, weight and waist and hip circumference were taken. In addition, body fat percentage using bioelectrical impedance and blood pressure were measured. Fasting blood samples were used for the determination of hemoglobin, HbA1c, glucose, lipid profile, alanine aminotransferase, aspartate aminotransferase, insulin, and creatinine levels. Within three months before surgery, a 2-h mixed meal tolerance test (MMT) was performed to assess insulin resistance and investigate dynamic alterations in circulating metabolites. The MMT consisted of two Nutridrink compact 125mL (Nutricia®), containing in total 23.3 grams fat, 74.3 grams carbohydrates (of which 38.5 grams sugar) and 24.0 grams protein. The participants received this meal after fasting for a minimum of nine hours. Time point zero refers to the moment at which the participant had fully consumed the meal. Blood samples were drawn *via* an intravenous line at baseline, 10, 20, 30, 60, 90 and 120 min. All samples were stored at −80°C until further processing.

#### Liver biopsies and histology

Liver histological sections were stained with Haematoxylin-Eosin and Sirius red and then reviewed by members of the Dutch Liver Pathology Panel after training sessions for NAFLD according to the Steatosis, Activity and Fibrosis (SAF) score (Bedossa et al., 2014). Difficult borderline cases were discussed during panel meetings for consensus. NAFLD was categorized into NAFL when steatosis was present in >5% of hepatocytes alone or with mild inflammation but without ballooning, or NASH when steatosis was present in >5% of hepatocytes and if ballooning and inflammation were both present in the biopsy. In the present study, no individuals were diagnosed with NASH based on histology.

#### Metabolome processing

EDTA plasma samples under fasting and two-hours after a MMT postprandial conditions were collected from 55 BARIA participants. All EDTA plasma samples were shipped to METABOLON (Morisville, NC, USA) for performing analysis using ultra high-performance liquid chromatography coupled to tandem mass spectrometry (LC-MS/MS) untargeted metabolomics. The metabolomic counts obtained, underwent significant curation via metabolites’ pre-filtering, imputation for subsets of metabolites’ missing values and data normalization, in order to minimize the effect of artifacts in the downstream analysis. Metabolomics prefiltering and imputation were performed by utilizing a variation of the Perseus platform (Koh et al., 2018). Essentially, data has been pre-filtered so as to have a maximum of 25% missing values for a metabolite across all samples. This was followed by a log transformation of all the measured metabolites’ raw intensities across the entire dataset. Then, we calculated the total data mean and standard deviation (by omitting missing values). Taking into account that the metabolite intensities distribution is approximately following normality, we chose a small distribution 2.5 standard deviations away from the original data mean towards the left tail of the original data distribution, with 0.5 standard deviations width. This new shrunken range corresponds to the actual lowest level of detection by the spectrometer. Here by drawing random values from this mini distribution, we fill the missing prefiltered data of choice.

Normalization was conducted to the total signal for each sample, since each sample is a separate injection on the mass spectrometer. Effective control for changes in sample matrix affects ionization efficiency, hence there can be inevitable differences in how much each sample is loaded onto the column with each injection, etc. Therefore, we summed up the total ion intensity (i.e. total signal) for each of the samples and identified the sample with the lowest total signal. After this we could proceed to calculating the correction factor for each sample:

$$\mathrm{CorrectionFacto}r_{i}=\frac{Total\;signal\;for\;each\;individual\;\mathrm{sampl}e_{i}}{Lowest\;total\;signal\;intensity}$$

Next, we divided each individual metabolite within a sample with the respective $\mathrm{CorrectionFacto}r_{i}\;$. Originally METABOLON measured 1345 metabolites, but after applying the above-mentioned methodology of imputation and normalization we included 988 metabolites for fasting metabolome and 1018 metabolites for postprandial metabolome.

#### Transcriptome processing

Biopsies from liver (55 samples), mesenteric adipose tissue (54 samples) and subcutaneous adipose tissue (55 samples) were collected at the time of the bariatric surgery. RNA was extracted from biopsies using TriPure Isolation Reagent (Roche, Basel, Switzerland) and Lysing Matrix D, 2 mL tubes (MP Biomedical, Irvine, CA, USA) in a FastPrep®-24 Instrument (MP Biomedical, Irvine, CA, USAs) with homogenization for 20 s at 4.0 m/s, with repeated bursts until no tissue was visible; homogenates were kept on ice for 5 min between homogenization bursts if multiple cycles were needed. RNA was purified with chloroform (Merck, Darmstadt, Germany) in phase lock gel tubes (5PRIME) with centrifugations at 4°C, and further purified and concentrated using the RNeasy MinElute kit (Qiagen, Venlo, The Netherlands). The quality of RNA was analysed on a BioAnalyzer instrument (Agilent), with quantification on Nanodrop (Thermo Fisher Scientific, Waltham, MA, USA). Due to degradation of the RNA, libraries for RNAseq sequencing were prepared by rRNA depletion; library preparation and sequencing were performed at Novogene (Nanjing, China) on an HiSeq instrument (Illumina Inc., San Diego, CA, USA) with 150 bp paired-end reads and 10G data/sample. The average read count per sample from liver was 42 ± 15 million. For mesenteric and subcutaneous adipose tissue, the average read count per sample were 43.2 ± 20 million. The extracted fastq files were analyzed with nf-core/rnaseq (Ewels et al., 2020), a bioinformatics analysis pipeline used for RNA sequencing data. The workflow processed raw data from FastQ inputs (FastQC, TrimGalore!), aligned the reads (STAR) with *Homo sapiens* GRCh38 as reference genome, generates gene counts (featureCounts, StringTie) and performed extensive quality-control on the results (RSeqQC, dupRadar, Preseq, edgeR, multiQC). The pipeline was built using Nextflow.

#### Microbiome processing

Fecal samples from 55 participants were collected on the day of surgery and immediately frozen at −80C. Total fecal genomic DNA was extracted from 100 mg of feces using a modification of the IHMS DNA extraction protocol Q (Costea et al., 2017). Briefly, fecal samples were extracted in Lysing Matrix E tubes (MP Biomedicals) containing ASL buffer (Qiagen, Venlo, The Netherlands), and lysis of cells was obtained, after homogenization by vortexing for 2 min, by two cycles of heating at 90°C for 10 min followed by three bursts of bead beating at 5.5 m/s for 60 s in a FastPrep®-24 Instrument (MP Biomedicals). After each bead-beating burst, samples were placed on ice for 5 min. The supernatants containing fecal DNA were collected after the two cycles by centrifugation at 4°C. Supernatants from the two centrifugations steps were pooled and a 600 μL aliquot from each sample was purified using the QIAamp DNA Mini kit (QIAGEN, Venlo, The Netherlands) in the QIAcube (QIAGEN Venlo, The Netherlands) instrument using the procedure for human DNA analysis. Samples were eluted in 200 μL of AE buffer (10 mmol/L Tris·Cl; 0.5 mmol/L EDTA; pH 9.0). Libraries for shotgun metagenomic sequencing were prepared by a PCR-free method; library preparation and sequencing were performed at Novogene (Nanjing, China) on an HiSeq instrument (Illumina Inc. San Diego, CA, USA) with 150 bp paired-end reads and 6G data/sample.

MEDUSA pipeline was used for pre-processing of raw shotgun metagenomics sequence data. MEDUSA is an integrated pipeline for analysis of short metagenomic reads, which maps reads to reference databases, combines output from several sequencing runs and manipulates tables of read counts. The input number of total reads from the metagenome analysis were on average 23.4 ± 2.2 million reads per sample and the total aligned reads 16.6 ± 1.8 million reads per sample. The sequencing runs had high quality with almost 98% of the reads passing the quality cut-off. Out of the high-quality reads, on average 0.04% aligned to the human genome, although the data had been cleaned for human reads. Out of the high quality non-human reads, 78.4% aligned to the MEDUSA’s software gene catalogue. Quality filtered reads were mapped to a genome catalogue and gene catalogue using Bowtie2 (Langmead and Salzberg 2012).

### Quantification and statistical analysis

Differential analysis of the plasma metabolome was conducted with two methods: ANOVA and Kruskal Wallis, with the use of HybridMTest package, that performs hybrid multiple testing using Empirical Bayes Probability (EBP). The cut-off significance level of p < 0.1 was used for identifying differentially significant metabolites with an adjusted EBP value.

Differential gene expression analysis for individuals with and without NAFL was performed for liver, subcutaneous adipose and mesenteric adipose tissues, respectively, in R with DESeq2 package (Love et al., 2014); log normalization is based on gene counts geometric distribution. The statistical analysis method for calculating differential expression rates is the Wald test. After False discovery rate (FDR) correction with multiple hypothesis testing with IHW package (Ignatiadis et al., 2016), we analyzed genes with p < 0.1 by DEGreport’s degPatterns function, to identify subgroups of co-expressed genes between individuals with and without NAFL. For these differentially significant co-expressed genes we performed gene enrichment with Enrichr platform (Chen et al., 2013) using KEGG metabolic pathways (Kanehisa and Goto 2000).

Statistical analysis on the metagenomics data was performed on rarefied count, (20 M reads per sample). The taxon ids were input to taxize package (Chamberlain and Szöcs 2014), to get full taxonomic information and ranking for the species. This dataset was input to DESeq2 and phyloseq packages (McMurdie and Holmes 2013) for conducting downstream differential statistical analysis. Similar to the BARIA transcriptomics counts, log normalization based on gene counts geometric distribution has been conducted with it. Statistical analysis methods for calculating differential expression rates was Wald Test. The IHW package, as part of DESeq2 suite, was utilized for multiple hypothesis testing and adjusting the respective p values, with alpha significance threshold set at p < 0.1.

Multi-omics integrative analysis has been conducted with DIABLO. DIABLO extends sparce Generalized Canonical Correlation Analysis (sGCCA), uses singular value decomposition for dimensionality reduction and selects co-expressed (correlated) variables that can explain the categorical outcome of interest (Tenenhaus et al., 2014), in our case non-NAFL or NAFL. DIABLO output a set of latent variables (components) based on the dimensionality of the input datasets. The chosen number of components could extract sufficient information to discriminate all phenotype groups. Then, a set of coefficients was attributed to each variable, that indicated the importance of each variable in DIABLO. The goal was to have maximization of the covariance between a linear combination of the variables from each input dataset and each categorical outcome. After tuning these two hyperparameters, DIABLO output a list of selected variables from each omic dataset, associated to each component, that could distinguish the given phenotypes.

## Data Availability

Of the individuals listed in the Table S1, high-dimensional data including fecal metagenomics, liver and adipose tissue transcriptomics is deposited in the European Nucleotide Archive (ENA PRJEB47902) and European Genome-Phenome Archive (EGAS00001005704) respectively. Code, plasma metabolomics and clinical data is available upon reasonable request. Any additional information required to reanalyze the data reported in this paper is available from the lead contact upon request.

## Supplemental information

Document S1. Figures S1–S5

Table S1. Differentially significant gut microbial species between individuals with and without NAFL padj: Wald test adjusted p value, as calculated by DESeq2, log2FoldChange: Fold change ratio, in log2 scale, between individuals without and with NAFL as related to STAR methods.

Table S2. Fasting Metabolomics statistical summary as reported in the STAR methods

Table S3 Post prandial metabolomics statistical summary as reported in the STAR methods

Table S4. Differentially significant genes in liver tissue between individuals with and without NAFL GeneName: Official Gene Symbol, GeneStableID: Ensemble Gene ID Identifier, pvalue: Wald test p value, as calculated by DESeq2, padj: Wald test adjusted p value, as calculated by DESeq2, log2FoldChange: Fold change ratio, in log2 scale, between individuals without and with NAFL as related to Figure 3

Table S5. Differentially significant genes in subcutaneous adipose tissue between individuals with and without NAFL GeneName: Official Gene Symbol, GeneStableID: Ensemble Gene ID Identifier, pvalue: Wald test p value, as calculated by DESeq2, padj: Wald test adjusted p value, as calculated by DESeq2, log2FoldChange: Fold change ratio, in log2 scale, between individuals without and with NAFL as related to Figure 3

Table S6. Differentially significant genes in mesenteric adipose tissue between individuals with and without NAFL GeneName: Official Gene Symbol, GeneStableID: Ensemble Gene ID Identifier, pvalue: Wald test p value, as calculated by DESeq2, padj: Wald test adjusted p value, as calculated by DESeq2, log2FoldChange: Fold change ratio, in log2 scale, between individuals without and with NAFL as related to Figure 3

Table S7. ID and NAFLD categories of individuals used in this study of whom high- dimensional data is available as reported in the STAR methods
