# Supplementary material for: A systems biology approach to study non-alcoholic fatty liver (NAFL) in women with obesity
Source: iScience. 2022 Aug 5;25(8):104828. doi: 10.1016/j.isci.2022.104828 (PMC9382345; doi:10.1016/j.isci.2022.104828)
Supplement: Document S1. Figures S1–S5 [file mmc1.pdf]

iScience, Volume *25*

Supplemental information

A systems biology approach to study non-alcoholic fatty liver (NAFL) in women with obesity

Abraham S. Meijnikman, Dimitra Lappa, Hilde Herrema, Omrum Aydin, Kimberly A. Krautkramer, Valentina Tremaroli, Louise E. Olofsson, Annika Lundqvist, Sjoerd Bruin, Yair Acherman, Joanne Verheij, Siv Hjorth, Victor E.A. Gerdes, Thue W. Schwartz, Albert K. Groen, Fredrik Bäckhed, Jens Nielsen, and Max Nieuwdorp

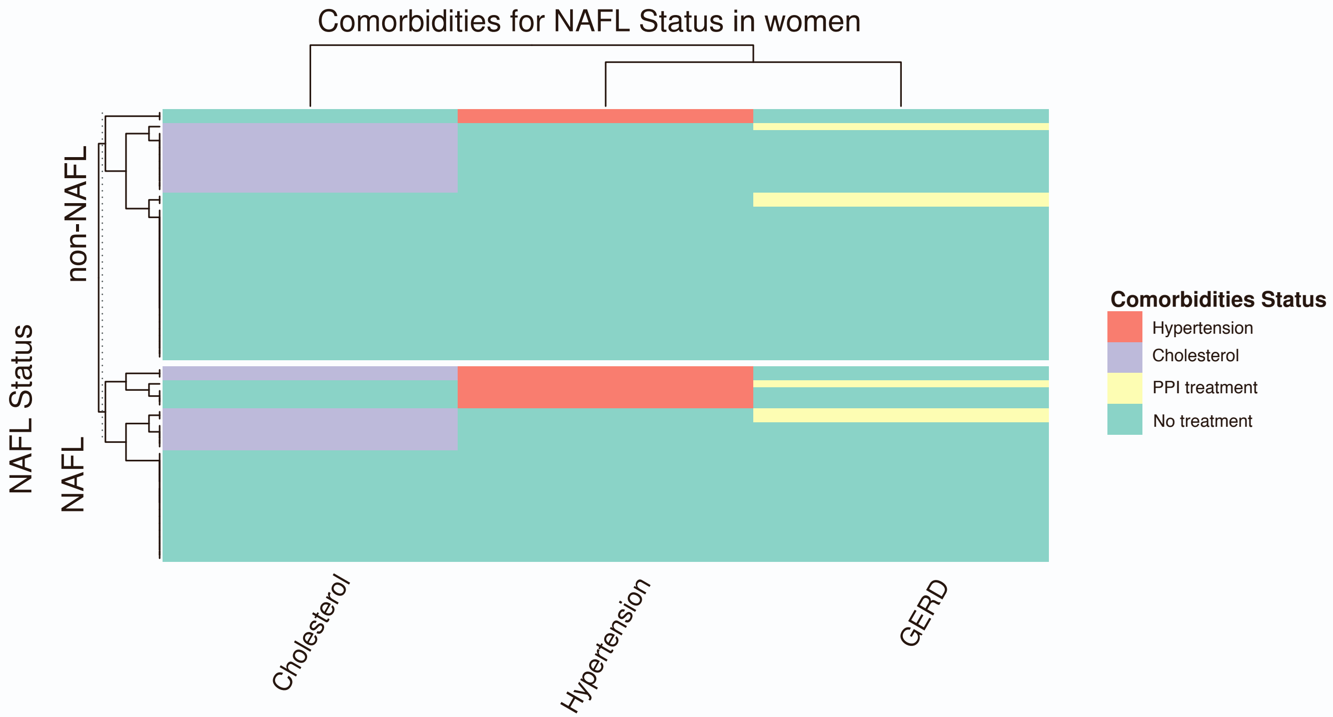

Figure S1. Distribution of treatments of cardiometabolic comorbidities,in individuals with and without NAFL.

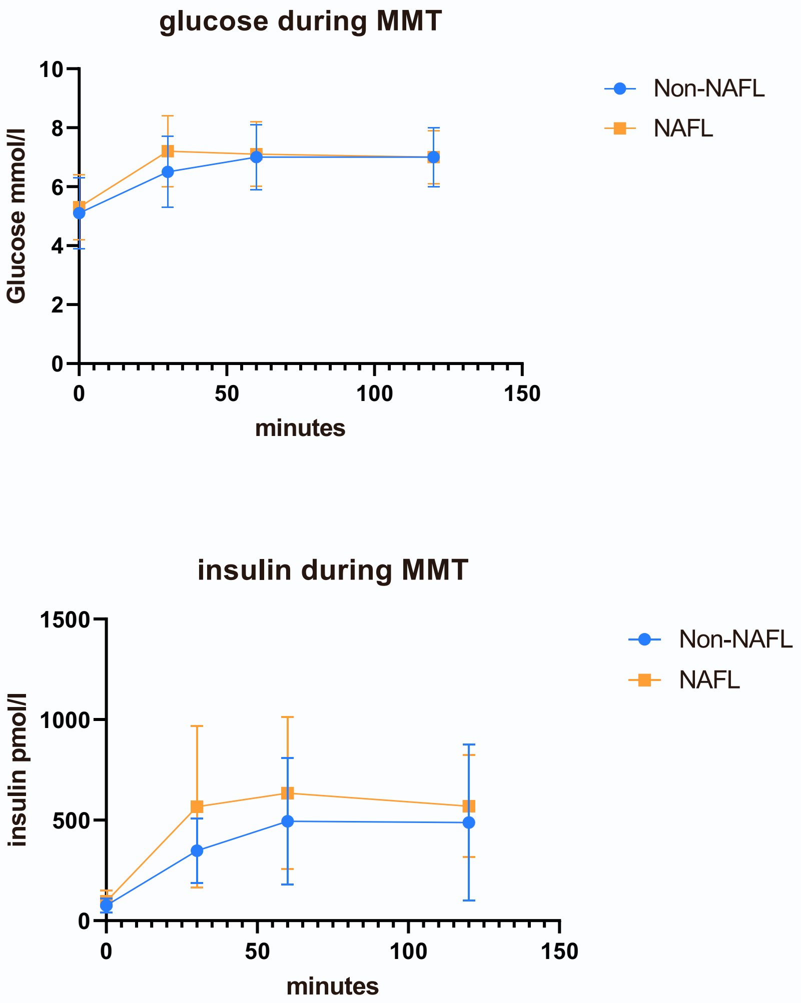

Figure S2. Glucose and insulin excursions during the mixed meal test in individuals with and without NAFL.

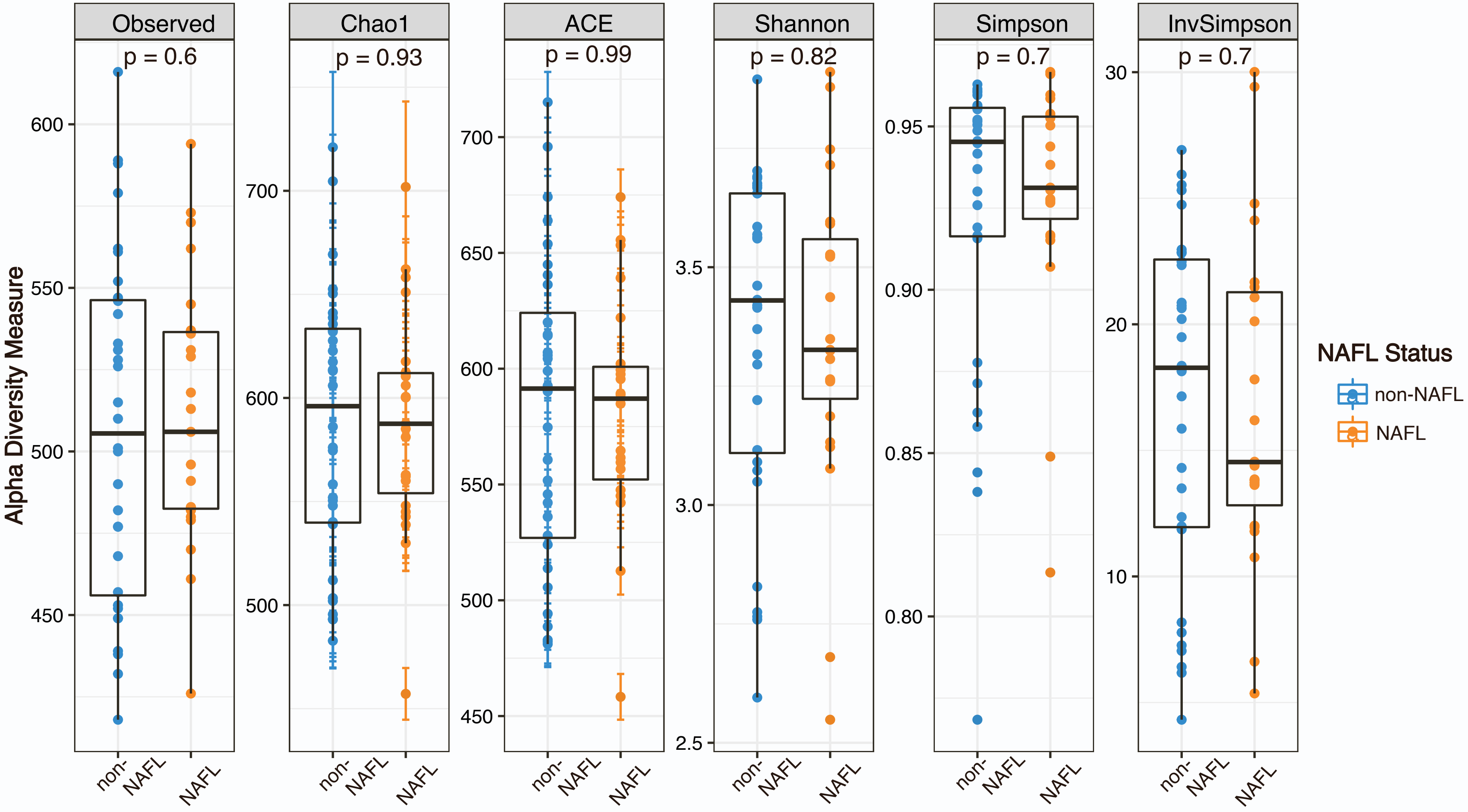

Figure S3. Different measures of gut microbial species alpha diversity

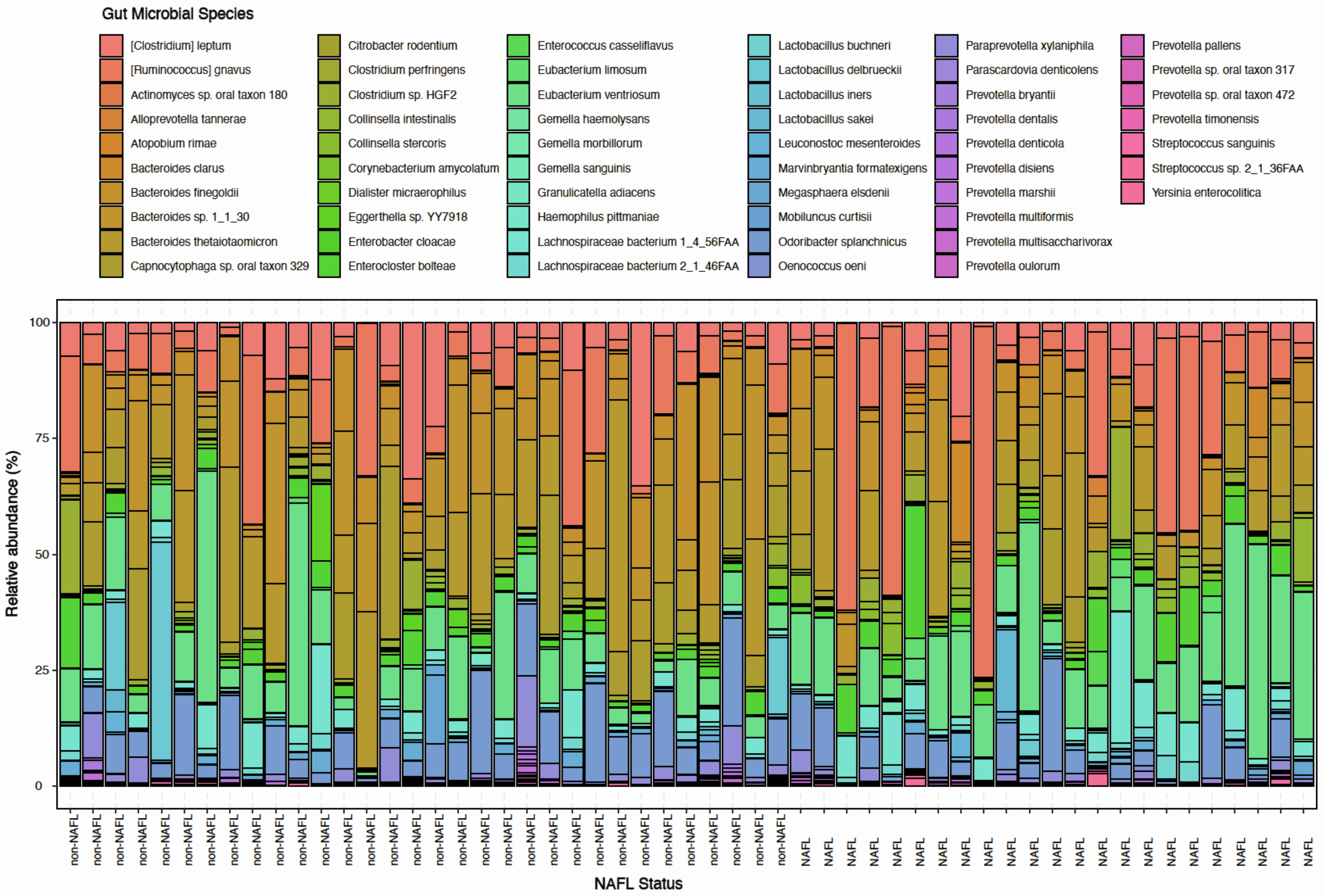

Figure S4. Relative abundance of differentially significant gut microbial species of each individuals with and without NAFL.

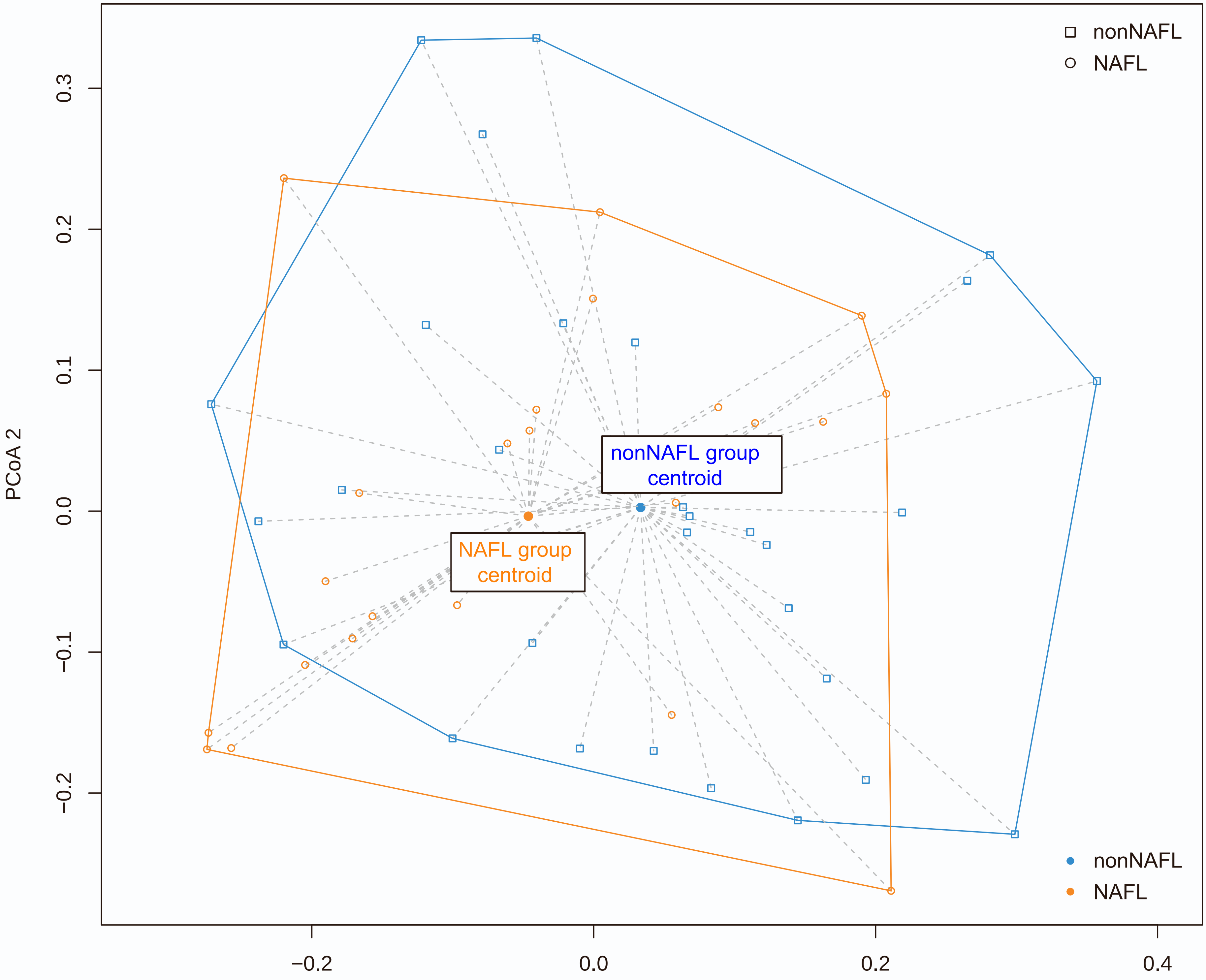

Figure S5. Beta dispersion Ordination Plots.

Supplementary Figure legends

Figure S1. Distribution of treatments of cardiometabolic comorbidities,in individuals with and without NAFL as related to Table 1.

Each line on the cluster heatmap denotes one individual and the individual's treatment status for hypertension, cholesterol, GERD and no cardiometabolic comorbidity treatment.

Figure S2. Glucose and insulin excursions during the mixed meal test in individuals with and without NAFL as related to Table 1.

Figure S3. Different measures of gut microbial species alpha diversity (Observed, Chao1, ACE, Shannon, Simpson, Inverse Simpson) for the 356 species identified in individuals with and without NAFL as related to STAR methods. FDR adjusted p was calculated with Wilcoxon Rank Sum test.

Figure S4. Relative abundance of differentially significant gut microbial species of each individuals with and without NAFL as related to STAR methods.

Figure S5. Beta dispersion Ordination Plots as related to Figure 3. a. Ordination plot of the groups and distances to centroids on the first two PCoA axes with a confidence interval of conf=0.90; b. Ordination plot of the groups and distances to centroids on the first two PCoA axes
